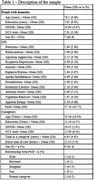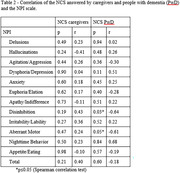# Investigation of the correlation between negative communication by caregivers and neuropsychiatric symptoms in people with dementia: Preliminary Results

**DOI:** 10.1002/alz.093009

**Published:** 2025-01-09

**Authors:** Lucimara Lehmen Gheno, Amanda Gorziza da Silva, Liana Lisboa Fernandez, Carlos Roberto Mello Rieder, Bárbara Costa Beber

**Affiliations:** ^1^ UFCSPA, porto alegre, Rio Grande do Sul Brazil; ^2^ Federal University of Health Sciences of Porto Alegre (UFCSPA), Viamão, Rio Grande do Sul Brazil; ^3^ Universidade Federal de Ciências da Saúde de Porto Alegre, Porto Alegre, Rio Grande do Sul Brazil; ^4^ Universidade Federal de Ciências da Saúde de Porto Alegre (UFCSPA), Porto Alegre, RS Brazil

## Abstract

**Background:**

Caregivers of people with dementia (PwD) often experience the burden of caregiving and depressive symptoms that may be associated. PwD who experience a higher occurrence of neuropsychiatric symptoms often cause a greater burden on their caregivers due to the difficulty in managing these symptoms. Caregivers may transfer their feelings of frustration and exhaustion with caregiving through the way they communicate with the PwD. On the other hand, it is also possible that inadequate communication triggers the occurrence of challenging behaviors in PwD, demonstrating the importance of communication between caregivers and care recipients. The objective of this study was to verify the correlation between the negative communication scale (NCS) answered by caregivers and PwD and the neuropsychiatric inventory (NPI).

**Method:**

This is a segment of a descriptive observational study with a quantitative approach, involving a sample of 11 dyads. The NPI and NCS were administered to the caregivers of PwD, and the NCS was also answered by the PwD themselves (to observe the perception of negative communication by the caregivers from the perspective of the PwD).

**Result:**

Characteristics of the sample are shown in Table 1. Correlations of NCS and NPI domains are described in Table 2 and show that the greater the disinhibition, the lower the perception of negative communication use by caregivers, as perceived by individuals with dementia. Also, the greater the aberrant motor changes, the lower the perception of negative communication use by caregivers, according to individuals with dementia.

**Conclusion:**

The results suggest a possible relationship between caregivers' communication and neuropsychiatric symptoms of PwD. Despite the presented correlations, it is necessary to increase the sample size to highlight/confirm these correlations.